# Heterogeneity of Hippo signalling activity in different histopathologic subtypes of renal cell carcinoma

**DOI:** 10.1111/jcmm.17632

**Published:** 2022-12-07

**Authors:** Nguyen Xuong Duong, Minh‐Khang Le, Tetsuo Kondo, Takahiko Mitsui

**Affiliations:** ^1^ Department of Urology University of Yamanashi Graduate School of Medical Sciences Chuo‐city Japan; ^2^ Department of Pathology University of Yamanashi Graduate School of Medical Sciences Chuo‐city Japan

**Keywords:** hippo signalling, oncogenic pathways, prognosis, renal cell carcinoma, TCGA, YAP1

## Abstract

This study aimed to reveal the prognostic role of the Hippo pathway in different histopathological subtypes of renal cell carcinoma (RCC). The TCGA‐KIRC (*n* = 537), TCGA‐KIRP (*n* = 291) and TCGA‐KICH (*n* = 113), which contain data about clear cell (ccRCC), papillary (pRCC) and chromophobe RCC (chRCC), respectively, were investigated. Gene Set Variation Analysis was used to compare the activity of many pathways within a single sample. Oncogenic pathway‐related expression differed between cases of ccRCC involving low and high Hippo pathway activity. There were two subsets of ccRCC, in which the cancer exhibited lower and higher Hippo signalling activity, respectively, compared with normal tissue. In the ccRCC cohort, lower Hippo pathway activity was associated with a higher clinical stage (*p* < 0.001). The Hippo pathway (HR = 0.29; 95% CI = 0.17–0.50, *p* < 0.001), apoptosis (HR = 6.02; 95% CI = 1.47–24.61; *p* = 0.013) and the p53 pathway (HR = 0.09; 95% CI = 0.02–0.36; *p* < 0.001) were identified as independent prognostic factors for ccRCC. The 5‐year overall survival of the ccRCC patients with low and high Hippo pathway activity were 51.9% (95% CI = 45.0–59.9) and 73.6% (95% CI = 67.8–79.9), respectively. In conclusion, the Hippo pathway plays an important role in the progression of ccRCC. Low Hippo pathway activity is associated with poor outcomes in ccRCC, indicating the tumour suppressor function of this pathway.

## 1 INTRODUCTION

Renal cell carcinoma (RCC) is one of three most common types of urological cancer, with approximately 431,288 new cases and 179,368 RCC‐related deaths occurring each year, following the most recent global data.^1^ RCC originates from the proximal tubular epithelium. Based on their morphological and cytogenetic characteristics, RCCs are classified into a wide range of histological subtypes,^2^ including three major types: clear cell (ccRCC), papillary (pRCC) and chromophobe (chRCC). In all RCC types, clinical stage and histopathology play key roles in prognosis. Augmentation in the percentage of incidental and localized tumours due to the development of imaging has improved the survival outcome of RCC.^3^, ^4^ Several risk factors such as tobacco exposure, hypertension, obesity^5^, ^6^ and an individual's genetic susceptibility affect the risk of RCC development. In addition, various genes and oncogenic pathways that may be related to RCC have been identified.^7^, ^8^

The Hippo signalling pathway is essential for regulating organ growth and regeneration.^9^, ^10^ It controls cell proliferation and apoptosis by negatively regulating the transcription of the co‐activators Yes‐associated protein (YAP) and transcriptional co‐activator with a PDZ‐binding domain (TAZ). Once the Hippo signalling is inhibited, an unphosphorylated form of the YAP/TAZ complex translocates to the nucleus. YAP/TAZ then binds to members of the transcriptional enhanced associate domain (TEAD) transcription factor family and elevates cell development and proliferation.^11^, ^12^ Interestingly, recent studies have found the relation between the Hippo–YAP/TAZ axis and the progression of some solid cancers, such as breast, liver, and lung cancer.^13^, ^14^, ^15^, ^16^ However, the role of the Hippo signalling pathway in RCC remains uncertain.

In this study, we aimed to evaluate the role of the Hippo signalling pathway in various histopathologic subtypes of RCC, specifically its expression and prognostic significance.

## 2 METHODS AND MATERIALS

### 2.1 The TCGA‐KIRC, TCGA‐KIRP and TCGA‐KICH projects

We investigated three RCC databases: TCGA‐KIRC (*n* = 537), TCGA‐KIRP (*n* = 291) and TCGA‐KICH (*n* = 113). The TCGA‐KIRC, TCGA‐KIRP and TCGA‐KICH contain data regarding ccRCC, pRCC and chRCC, respectively. We extracted gene expression data from these databases with the following filter criteria: (1) data type: gene expression quantification, (2) experimental strategy: RNA‐seq and (3) workflow type: STAR – Counts. The total number of collected files was 1026, which included gene expression files for normal tissue samples. After eliminating the files relating to normal tissue samples, we were left with 888 files, each of which corresponded to a clinical case. Therefore, there were 53 cases without expression data. The clinical data of the three cohorts were also retrieved, including age, sex, race, American Joint Cancer Committee (AJCC) stage, overall survival time and vital status.

### 2.2 Gene Set Variation Analysis

Gene set variation analysis (GSVA) is a special type of single‐sample gene set enrichment analysis (ssGSEA) based on the principle of conventional GSEA.^17^ However, instead of comparing the activity of pathways with different phenotypes (such as tumoral and normal phenotypes), the ssGSEA compares the activity of many pathways within a single sample. Therefore, in ssGSEA the enrichment score (ES) of a pathway can be understood as its intrinsic dynamic level relative to the basal expression activity. Therefore, interpreting the ssGSEA data of a pathway requires taking the absolute counts of its member genes into account. For a given pathway, a strong Pearson (parametric) correlation between its ES and the absolute counts of its member genes supports the ability of the ES to reflect the pathway's true activity level. GSVA was developed by many authors.^18^, ^19^ We utilized the GSVA package, version 1.44.2, to conduct this experiment.

### 2.3 Statistical analyses

We reported continuous variables as mean and standard deviation values and categorical variables as the number of cases and percentages. The Wilcoxon and Kruskal–Wallis tests were used to compare continuous variables, whereas the chi‐squared test was used to compare categorical parameters. We conducted correlation analyses using Pearson's method. Hypothesis tests were considered significant when *p* < 0.05. All analyses were performed using the R software, version 4.2.1 (The R Foundation). Heatmap plots were drawn using the bioinfokit package, version 2.0.8^20^ with Python, version 3.9.13.

## 3 RESULTS

### 3.1 Clinical characteristics of the cohorts

Table 1 shows the differences in the clinical parameters of the three cohorts. The distributions of age, sex and race differed among the ccRCC, pRCC and chRCC patients. However, these variables exhibited similar tendencies in all three cohorts. All types of RCC mainly occurred in the elderly and males. The distribution of race was difficult to interpret due to selection bias. TNM categories and AJCC stages also differed among the three cohorts. Stage I RCC was the most common type in all three cohorts, followed by stage III (TCGA‐KIRC and TCGA‐KIRP) or stage II (TCGA‐KICH).

**TABLE 1:** Clinical characteristics of the three cohorts

Variables	TCGA‐KIRC (*n* = 537)	TCGA‐KIRP (*n* = 291)	TCGA‐KICH (*n* = 113)	*p*‐Value
Age (years)	60.6 (±14.3)	61.4 (±12.0)	51.5 (±14.3)	0.027*
Sex				
Female	187 (35.2%)	76 (26.2%)	27 (40.9%)	0.011*
Male	345 (64.8%)	214 (73.8%)	39 (59.1%)	
Race				
Caucasian	461 (87.8%)	206 (74.9%)	58 (90.7%)	<0.001*
Non‐Caucasian	64 (12.2%)	69 (25.1%)	6 (9.3%)	
T stage				
T1	272 (51.1%)	193 (66.6%)	21 (31.8%)	<0.001*
T2	69 (13.0%)	33 (11.4%)	25 (37.9%)	
T3	180 (33.8%)	60 (20.7%)	18 (27.3%)	
T4	11 (2.1%)	2 (0.7%)	2 (3.0%)	
Tx	0 (0.0%)	2 (0.7%)	0 (0.0%)	
N stage				
N0	240 (45.1%)	50 (17.3%)	40 (60.6%)	<0.001*
N1	16 (3.0%)	24 (8.3%)	3 (4.5%)	
N2	0 (0.0%)	4 (1.4%)	2 (3.0%)	
Nx	276 (51.9%)	211 (73.0%)	21 (31.8%)	
M stage				
M0	421 (79.4%)	95 (34.5%)	34 (75.6%)	<0.001*
M1	79 (14.9%)	9 (3.3%)	2 (4.4%)	
Mx	30 (5.7%)	171 (62.2%)	9 (20%)	
AJCC stage				
I	266 (50.3%)	172 (66.2%)	21 (31.8%)	<0.001*
II	57 (10.8%)	21 (8.1%)	25 (37.9%)	
III	123 (23.3%)	52 (20.0%)	14 (21.2%)	
IV	83 (15.7%)	15 (5.8%)	6 (9.1%)	
Overall survival (months)	44.4 (±32.7)	35.1 (±29.9)	71.1 (±40.6)	0.039*

* Significant values.

### 3.2 GSVA results can reflect the true activity of the Hippo signalling pathway

To investigate the activity of signalling pathways, we performed GSVA of all available samples from the three cohorts (*n* = 888). The results yielded ES for each sample and each pathway. To examine whether the ES of the Hippo pathway was closely related to the corresponding absolute counts of the pathway's member genes, we performed analyses of the correlations between the ES of the Hippo pathway and the absolute counts of its 20 member genes according to the MSigDB database (www.gsea‐msigdb.org): *DVL2*, *WWTR1*, *STK4*, *TJP1*, *STK3*, *YWHAE*, *WWC1*, *AMOTL2*, *MOB1A*, *TJP2*, *AMOT*, *LATS1*, *NPHP4*, *YAP1*, *LATS2*, *SAV1*, *CASP3*, *AMOTL1*, *YWHAB* and *MOB1B*. Table 2 summarizes the results, which showed that there were significant positive correlations between the ES of the Hippo pathway and the absolute counts of almost all of its member genes.

**TABLE 2:** Pearson's correlation analyses between enrichment score of Hippo pathway and its member genes

Gene	Coefficient	95% CI	*p*‐Value
*AMOT*	0.57	0.53–0.62	<0.001*
*AMOTL1*	0.64	0.60–0.67	<0.001*
*AMOTL2*	0.41	0.36–0.47	<0.001*
*CASP3*	0.13	0.07–0.20	<0.001*
*DVL2*	0.11	0.05–0.18	<0.001*
*LATS1*	0.62	0.57–0.66	<0.001*
*LATS2*	0.55	0.50–0.60	<0.001*
*MOB1A*	0.5	0.45–0.55	<0.001*
*MOB1B*	0.03	−0.13	0.426
*NPHP4*	0.15	0.08–0.21	<0.001*
*SAV1*	0.39	0.33–0.44	<0.001*
*STK3*	0.23	0.16–0.29	<0.001*
*STK4*	0.23	0.17–0.29	<0.001*
*TJP1*	0.62	0.58–0.66	<0.001*
*TJP2*	0.13	0.07–0.20	<0.001*
*WWC1*	0.22	0.16–0.28	<0.001*
*WWTR1*	0.51	0.46–0.56	<0.001*
*YAP1*	0.53	0.48–0.57	<0.001*
*YWHAB*	0.58	0.54–0.62	<0.001*
*YWHAE*	0.31	0.25–0.37	<0.001*

Abbreviation: 95% CI, 95% confidence interval.

* Significant values.

### 3.3 Enrichment levels of the Hippo pathway were associated with oncogenic pathways in ccRCC

To examine whether different Hippo pathway activity levels are related to different oncogenic pathway landscapes, we conducted a two‐stage analysis. The first stage involved the unsupervised clustering of Hippo ES to identify different activity level groups for this pathway. The second stage involved dimensionality reduction of the ES of the 50 hallmark pathways in the MSigDB database. The purpose of this analysis was to test and visualize whether different ES groups can be delineated in the embedded plane. If clear visual delineation is seen, we can conclude that the activity of the Hippo pathway is associated with different oncogenic landscapes. This two‐stage analysis was performed separately for each RCC subtype due to their biological and oncogenic differences.

**FIGURE 1:**
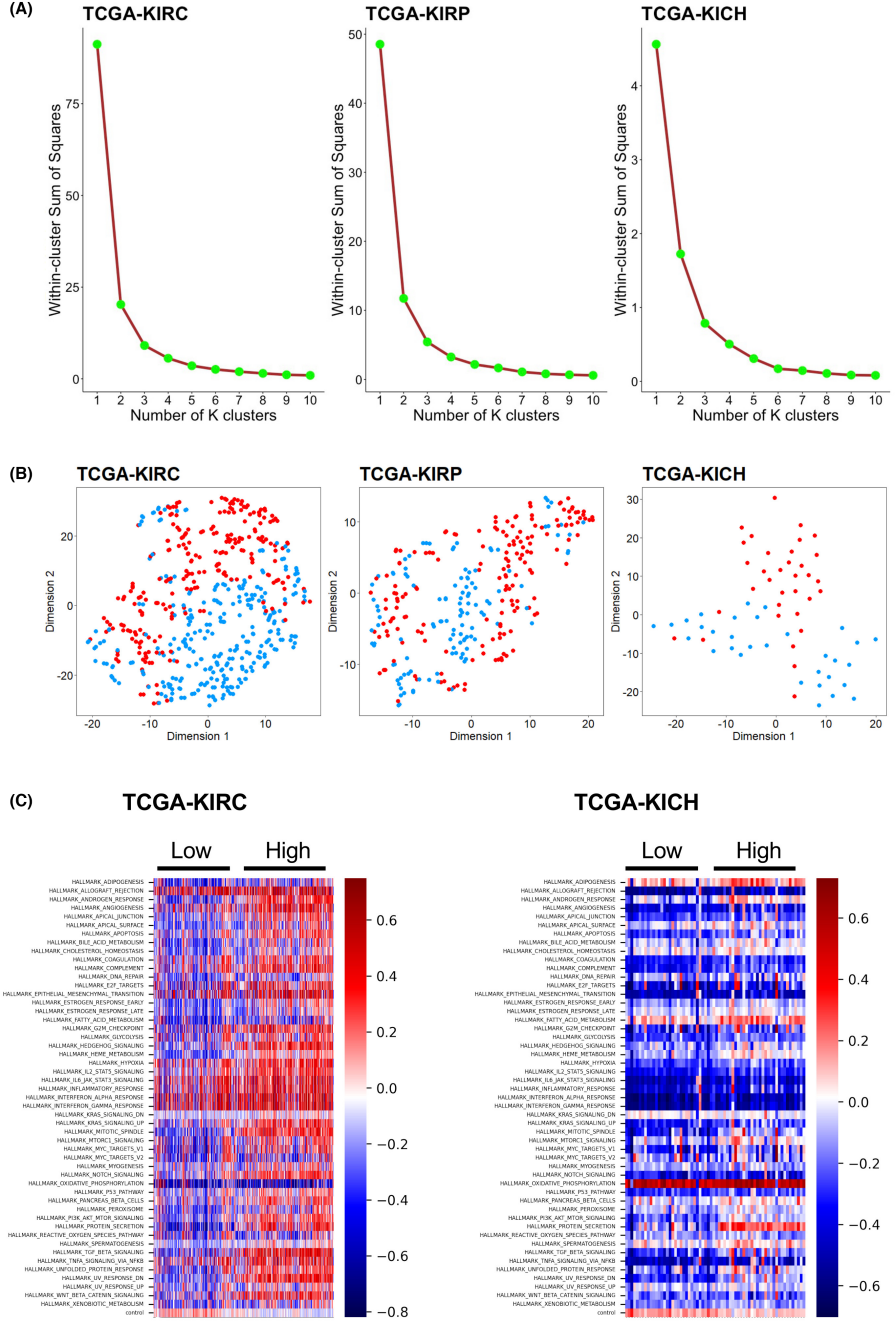
Enrichment levels of the Hippo pathway in each RCC subtype. (A) K‐means clustering analyses in TCGA‐KIRC (ccRCC), TCGA‐KIRP (pRCC) and TCGA‐KICH (chRCC) cohorts. t‐SNE dimension reduction was performed to embed enrichment score (ES) of 50 hallmark pathways into a two‐dimensional space. (B) Data points for the Hippo pathway ES clusters of the TCGA‐KIRC (ccRCC), TCGA‐KIRP (pRCC) and TCGA‐KICH (chRCC) cohorts. (C) Heatmaps of the TCGA‐KIRC (ccRCC) and TCGA‐KIRP (pRCC) cohorts showing the relationships between Hippo pathways and other oncogenic pathways.

In the first stage, we grouped the Hippo ES for each cohort by K‐means clustering analyses. The Elbow method was used to select the optimal K‐values for each cohort, based on the within‐cluster sum of squares (WSS) (Figure 1A). Since the WSS dropped significantly from K = 1 to K = 2 in all cohorts, we selected K = 2 in all cohorts. We then performed t‐SNE dimension reduction in the second stage to embed the ES for the 50 hallmark pathways into a two‐dimensional space. The data points were then colour‐coded based on the Hippo ES clusters identified in the first stage. Figure 1B shows how well these groups can be delineated in the embedded space. In the TCGA‐KIRC and TCGA‐KICH t‐SNE plots, the low‐ES and high‐ES points could be grouped. However, the low‐ES and high‐ES points in the TCGA‐KIRP plots seemed to be scattered randomly. In addition, we created heatmaps to examine the relationships between Hippo signalling and other hallmark oncogenic pathways in the TCGA‐KIRC and TCGA‐KICH cohorts (Figure 1C).

### 3.4 Comparison of Hippo signalling activity between paired normal and tumoral tissue samples in low and high Hippo signalling activity subgroups

**FIGURE 2:**
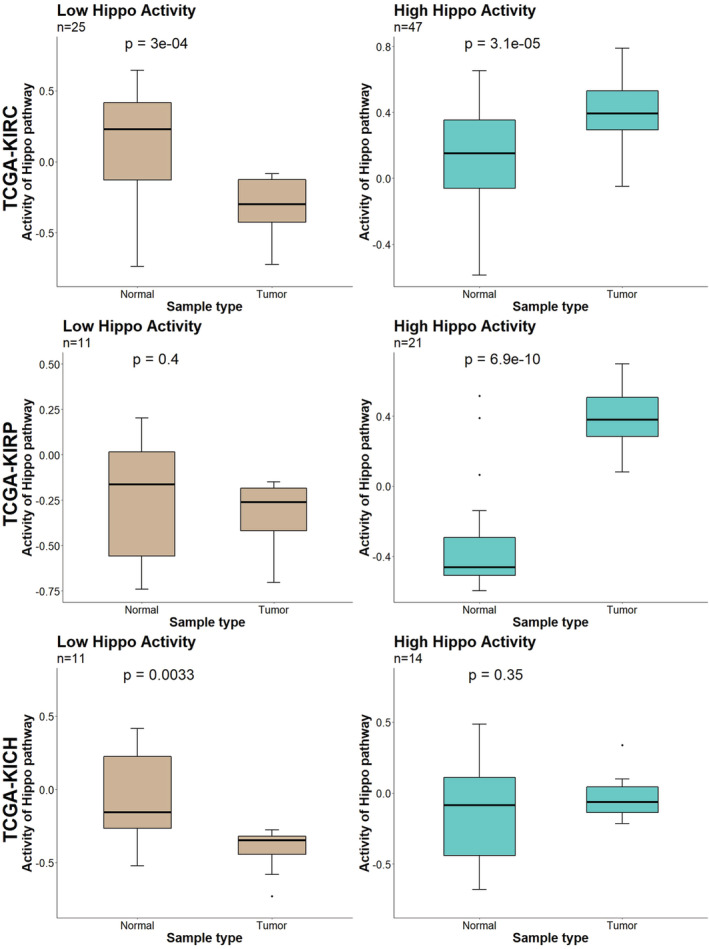
Comparisons of Hippo signalling activity between tumoral and paired normal tissue samples in the TCGA‐KIRC (ccRCC), TCGA‐KIRP (pRCC) and TCGA‐KICH (chRCC) cohorts.

To compare the observed Hippo pathway activity of the tumours with that of paired normal tissue samples, we performed GSVA of all the available paired normal tissue samples. As paired normal tissue samples were not available for all patients in the databases, we only analysed cases for which both normal and tumour tissue samples were available in this experiment. Figure 2 illustrates the results we obtained using paired two‐sample *t*‐tests in the low and high Hippo signalling activity subgroups. The boxplots of the TCGA‐KIRC cohort show a bimodal Hippo activity pattern for the tumoral samples compared with the activity of the normal tissue samples, which was not seen in the TCGA‐KIRP or TCGA‐KICH cohorts. These results can be interpreted as follows: (1) A subset of pRCC tumours exhibit higher Hippo signalling activity, while (2) a subset of chRCC tumours demonstrate lower Hippo signalling activity compared with the levels seen in normal tissue; (3) however, there are two biologically distinct subsets of ccRCC (as shown in Figure 1), which show lower and higher Hippo activity levels, respectively, than normal tissue.

### 3.5 Lower Hippo pathway activity was related to worse prognoses in ccRCC

**FIGURE 3:**
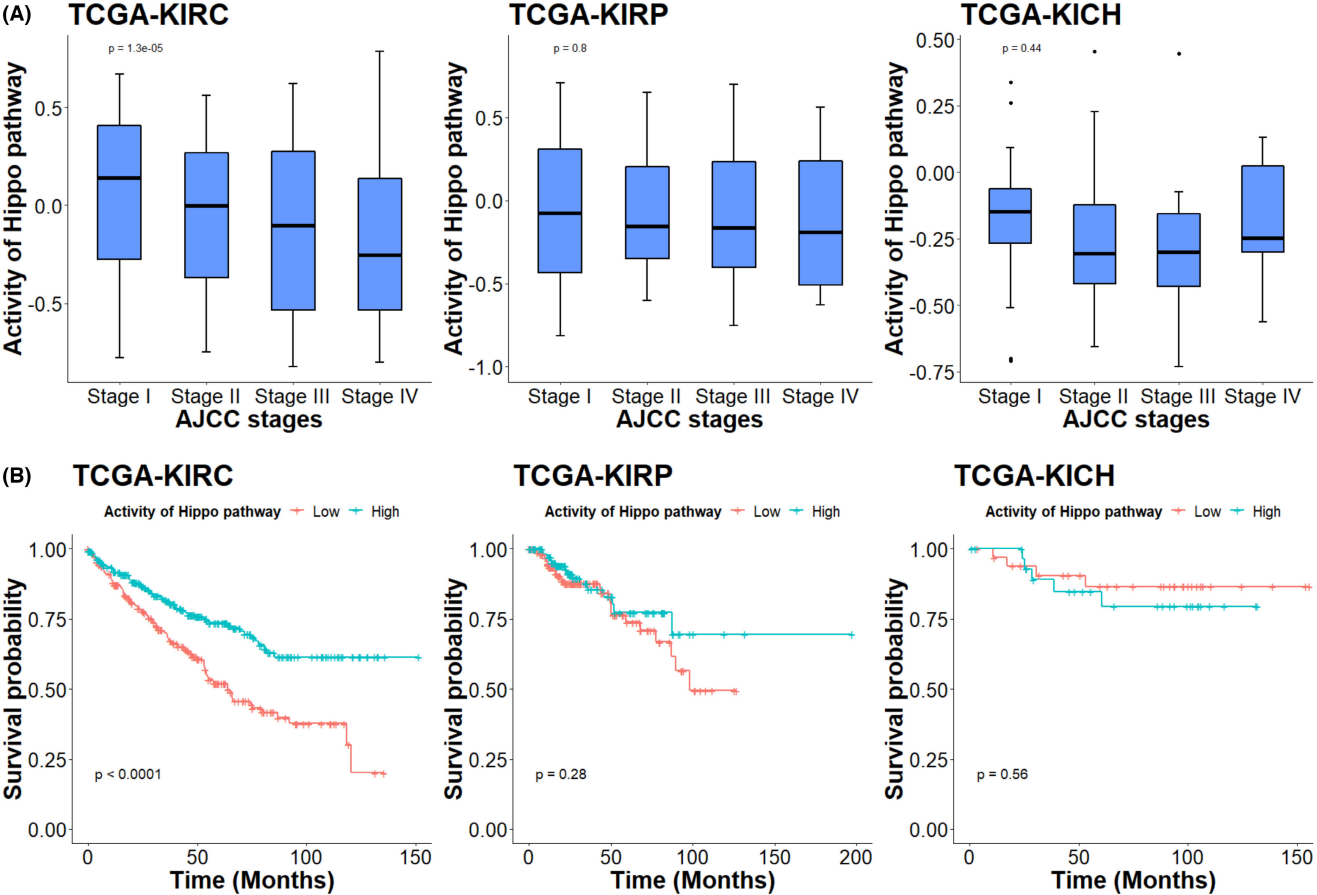
(A) Hippo signalling activity in different AJCC stage; (B) Kaplan–Meier analyses compairing tumours with low and high Hippo pathway activity in TCGA‐KIRC (ccRCC), TCGA‐KIRP (pRCC) and TCGA‐KICH (chRCC) cohorts.

Figure 3A shows the activity of the Hippo signalling pathway in different AJCC stages. In the TCGA‐KIRC cohort, tumours with higher stages had lower Hippo signalling pathway activity (*p* < 0.001). However, there were no such differences in the other two cohorts. To investigate the prognostic value of the Hippo pathway, we performed Kaplan–Meier (K‐M) analyses comparing tumours with low and high Hippo signalling pathway activity (Figure 3B). Lower Hippo signalling pathway activity was only associated with a worse prognosis in the TCGA‐KIRC cohort (*p* < 0.001). To further validate the significant results of the K‐M analysis in the TCGA‐KIRC patients, we performed univariate and multivariate Cox proportional hazards analyses to examine the prognostic value of the Hippo pathway and other relevant oncogenic pathways in ccRCC (Table 3). The other examined pathways included apoptosis,^21^ epithelial‐mesenchymal transition (EMT),^22^, ^23^ the p53 pathway,^24^ DNA repair, angiogenesis,^25^ the inflammatory response,^26^ hypoxia^27^ and the mTORC1 pathway.^28^ We also calculated the basal gene expression level as the mean expression level of all genes in each sample. This value was used to statistically control for the potential relativity effect of GSVA. The multivariate analysis only included pathways that were found to be significant in the univariate phase. As a result, the Hippo signalling pathway (hazard ratio [HR] = 0.37; 95% confidence interval [CI] = 0.24–0.56, *p* < 0.001), apoptosis (HR = 5.79; 95% CI = 1.39–24.18; *p* = 0.016) and the p53 pathway (HR = 0.09; 95% CI = 0.02–0.46; *p* = 0.003) were identified as independent prognostic factors for ccRCC. The 5‐year overall survival of the low and high Hippo pathway activity groups of the TCGA‐KIRC cohorts were 51.9% (95% CI = 45.0–59.9) and 73.6% (95% CI = 67.8–79.9), respectively.

**TABLE 3:** Univariate and multivariate Cox hazard analyses of signalling pathways

	Univariate		Multivariate	
Signalling pathways	HR (95% CI)	*p*‐Value	HR (95% CI)	*p*‐Value
Hippo	0.37 (0.26–0.53)	<0.001*	0.37 (0.24–0.56)	<0.001*
Apoptosis	0.41 (0.22–0.74)	0.003*	5.79 (1.39–24.18)	0.016*
EMT	0.92 (0.61–1.39)	0.681		
p53	0.24 (0.12–0.49)	<0.001*	0.09 (0.02–0.46)	0.003*
DNA repair	0.74 (0.38–1.45)	0.382		
Angiogenesis	0.51 (0.32–0.80)	<0.001*	0.87 (0.39–1.91)	0.72
Inflammatory response	0.74 (0.45–1.20)	0.215		
mTORC1	0.66 (0.40–1.11)	0.115		
Hypoxia	0.39 (0.23–0.68)	<0.001*	1.00 (0.32–3.09)	0.99
Base gene expression^a^	1.18 (1.06–1.30)	0.002*	0.90 (0.77–1.05)	0.172

Abbreviations: 95% CI, 95% confidence interval; EMT, epithelial‐mesenchymal transition; GSVA, gene set variation analysis; HR, hazard ratio.

^a^ Base gene expression is summarized simply as the mean expression level of all genes in each sample to control the relativity effects of GSVA.

* Significant values.

## 4 DISCUSSION

Renal cell carcinoma is a common type of urological cancer, with an incidence of 2%–3% of total adult cancers. It primarily presents in elderly patients, aged 55–75 years, with a male predominance.^29^ In the present study, all RCC types were also mainly developed in elderly and male patients.

The core components of Hippo signalling include the mammalian sterile 20‐like kinase 1/2 (MST1/2), large tumour suppressor 1/2 (LATS1/2) and Slvador homologue 1 (SAV1). The MST1/2 in complex with protein SAV1 phosphorylates LATS1/2. LATS1/2 in cooperation with the regulator protein MOB1^30^ phosphorylates and prevents YAP/TAZ from undergoing nuclear translocation. The phosphorylated YAP/TAZ complex loses its transcriptional co‐activator function, which contributes to cell proliferation as well as the suppression of apoptosis.^31^ The inhibition of Hippo signalling or any of its core components results in increasing cell proliferation and decreasing apoptosis.^32^, ^33^, ^34^

Studies found that YAP/TAZ plays a role as an oncoprotein in numerous carcinomas.^35^, ^36^, ^37^ Inactivation of the YAP/TAZ complex by the Hippo pathway performs a crucial role in the regulation of tissue growth and oncogenic suppression.^33^, ^38^ However, the role of dysregulated Hippo signalling in the pathogenesis of RCC remains unclear. Our recent study showed that Hippo signalling activity is related to different oncogenic pathways in the ccRCC. In detail, low Hippo pathway activity corresponds with the downregulation of almost the Hallmark pathways.

The Hippo signalling interacts with oncogenic pathways in response to a variety of stress stimuli such as hypoxia, energy metabolism and reactive oxygen species.^39^ Recent studies demonstrated that renal tubular epithelial cells were generated by *Sav1* deficiency.^40^ In high‐grade ccRCC, the downregulation of SAV1 and the consequent YAP activation encouraged cell growth and prevented apoptosis.^41^, ^42^ Both permanent and impermanent hypoxia microenvironments, which are caused by tumour growth, involve the elevation of cell proliferation and/or the inhibition of apoptosis. The tumour cells in these hypoxic microenvironments undergo an adaptive transcription response mediated by hypoxia‐inducible factors (HIFs),^43^, ^44^ which transcriptionally upregulates oncogenic hypoxia‐responsive genes, including vascular endothelial growth factor A (VEGFA).^45^, ^46^ The relationships between the HIFs and Hippo pathway in the hypoxia‐induced adaptive response have been clarified in a previous study.^47^

Based on comparisons of Hippo signalling activity levels between tumour tissue and normal tissue, we found that the ccRCC patients could be seperated into two subsets with different biological and prognostic characteristics. Previous studies that evaluated the expression of the core components in the Hippo pathway and its target proteins, YAP and TAZ, found contradictory results.^48^, ^49^, ^50^, ^51^ The majority of these researches indicated that disruption of the Hippo signalling pathway results in the overexpression of the target proteins,^49^, ^50^, ^51^ YAP and TAZ, in ccRCC. However, Hu X et al.^48^ showed that YAP protein expression is downregulated in these patients. In our study, we found that there are two distinct subsets of ccRCC with different relative Hippo pathway activity levels compared with normal tissue.

In RCC, histological, clinical and molecular factors are three main prognostic factors.^52^, ^53^ A prognostic factor supplies information regarding disease outcomes, especially cancer outcomes, irrespective of the treatment received.^54^ To further investigate the prognostic impact of Hippo pathway activity in ccRCC, K‐M analysis comparing the tumours with low and high Hippo signalling activity was performed. We found that ccRCC with low Hippo signalling activity exhibited a worse prognosis. The 5‐year overall survival of the TCGA‐KIRC cohort patients with low Hippo pathway activity was significantly lower than that of those with high Hippo pathway activity. The present study provides evidence of the anti‐oncogenic function of the Hippo pathway. In the multivariate analyses of Hippo and other oncogenic pathways in ccRCC, the Hippo and p53 pathways were identified as tumour suppressor pathways. These two pathways work in concert on multiple levels to protect genome integrity in response to DNA damage.^55^ Loss of this coordination facilitates disorders, oncogenesis and tissue overgrowth.

Papillary and chromophobe are the other two main subtypes of RCC. Papillary RCC, the second most prevalent histological subtype of RCC, from 10%–15% of cases,^2^ has a propensity towards multicentricity, and is more common in individuals with end‐stage renal disease and acquired renal cystic disease.^56^ Chromophobe RCC, a distinct histologic subtype of RCC that makes up 3%–5% of all RCC, has originated from the distal convoluted tubules.^2^, ^56^, ^57^ No significant differences in the activity levels of the oncogenic pathways were found between the two Hippo pathway activity groups in the pRCC or chRCC cohorts. In addition, the pRCC and chRCC tissue seemed to exhibit higher and lower Hippo signalling activity, respectively, than normal tissue.

There are several common genetic mutations in RCC.^58^ They include *VHL*, *PBRM1*, *SETD2*, *BAP1*, *PTEN*, *MTOR* and *PIK3CA* in ccRCC; the *MET*, *PBRM1*, *SETD2* and *BAP1* in pRCC; the *TP53* and *PTEN* in chRCC. We investigated these genetic alterations in three subtypes of RCC (Table S1). However, there was no significant difference in genetic alterations between the high and low Hippo groups in both three subtypes. A further investigation focusing on two subtypes pRCC and chRCC would be of great interest.

In addition to histological and clinical prognostic factors, which have been used in previous studies and have great predictive abilities, the discovery of molecular markers has provided new understanding into RCC biology and aided the development of novel targeted therapies. A number of proliferation biomarkers, including p53, Ki‐67 and PTEN, along with factors associated with the HIF pathway have been researched.^59^, ^60^ Molecular marker research faces numerous challenges, including poor sample collection, issues with quantity and quality, a lack of clinical data as well as concerns regarding routine clinical applicability. The use of genomic, transcriptomic and proteomic signatures in biomolecular approaches for outcome prediction remains at an early phase.

This study was affected by selection bias, as it was based on public databases. In addition, the role of Hippo signalling in the progression of RCC remains obscure. However, the present study of the role and prognostic impact of the Hippo signalling pathway in RCC will provide researchers with insights into the mechanisms of tumour progression and aid future studies of RCC.

## 5 CONCLUSION

Our study provided persuasive evidence that the Hippo pathway is associated with oncogenic pathways and plays an important role in the progression of ccRCC. Low Hippo signalling activity is an indicator of poor clinical outcomes in ccRCC. Taken together, Hippo signalling appears to function as a tumour suppressor pathway.

## AUTHOR CONTRIBUTIONS

**Nguyen Xuong Duong:** Conceptualization (equal); data curation (equal); formal analysis (supporting); investigation (equal); methodology (equal); project administration (equal); validation (equal); visualization (equal); writing – original draft (equal); writing – review and editing (equal). **Minh‐Khang Le:** Conceptualization (equal); data curation (equal); formal analysis (equal); investigation (equal); methodology (equal); visualization (equal); writing – original draft (equal); writing – review and editing (equal). **Tetsuo Kondo:** Data curation (equal); formal analysis (equal); methodology (equal); project administration (equal); supervision (equal); validation (equal); visualization (equal). **Takahiko Mitsui:** Conceptualization (equal); methodology (equal); project administration (equal); supervision (equal); validation (equal); visualization (equal); writing – review and editing (equal).

## CONFLICT OF INTEREST

The authors declare no conflicts of interest.

## Supporting information


Table S1.

## Data Availability

The data that support the findings of this study are available in Genomic Data Commons Data Portal at https://portal.gdc.cancer.gov.
