# Supplementary material for: Heterogeneity of Hippo signalling activity in different histopathologic subtypes of renal cell carcinoma
Source: J Cell Mol Med. 2022 Dec 7;27(1):66–75. doi: 10.1111/jcmm.17632 (PMC9806300; doi:10.1111/jcmm.17632)
Supplement: Supplementary file 1 — Table S1. [file JCMM-27-66-s001.docx]

**Supplementary table:** Genetic alterations comparison between 3 subgroups of RCC

| **Genes mutation** | | **TCGA-KIRC** | | |  | **TCGA-KIRP** | | |  | **TCGA-KICH** | | |
| --- | --- | --- | --- | --- | --- | --- | --- | --- | --- | --- | --- | --- |
|  |  | **High** | **Low** | **p-value** |  | **High** | **Low** | **p-value** |  | **High** | **Low** | **p-value** |
| VHL | No | 202 (69.2%) | 177 (73.8%) | 0.250 |  | 120 (99.2%) | 166 (98.2%) | 0.643 |  | 32 (100.0%) | 34 (100.0%) | 1.000 |
|  | Yes | 90 (30.8%) | 63 (26.2%) |  |  | 1 (0.8%) | 3 (1.8%) |  |  | 0 (0.0%) | 0 (0.0%) |  |
| MET | No | 288 (98.6%) | 239 (99.6%) | 0.385 |  | 108 (89.3%) | 162 (95.9%) | 0.035* |  | 32 (100.0%) | 34 (100.0%) | 1.000 |
|  | Yes | 4 (1.4%) | 1 (0.4%) |  |  | 13 (10.7%) | 7 (4.1%) |  |  | 0 (0.0%) | 0 (0.0%) |  |
| FH | No | 292 (100.0%) | 240 (100.0%) | 1.000 |  | 120 (99.2%) | 168 (99.4%) | 1.000 |  | 32 (100.0%) | 34 (100.0%) | 1.000 |
|  | Yes | 0 (0.0%) | 0 (0.0%) |  |  | 1 (0.8%) | 1 (0.6%) |  |  | 0 (0.0%) | 0 (0.0%) |  |
| FLCN | No | 291 (99.7%) | 239 (99.6%) | 1.000 |  | 119 (98.3%) | 168 (99.4%) | 0.573 |  | 32 (100.0%) | 34 (100.0%) | 1.000 |
|  | Yes | 1 (0.3%) | 1 (0.4%) |  |  | 2 (1.7%) | 1 (0.6%) |  |  | 0 (0.0%) | 0 (0.0%) |  |
| PTEN | No | 284 (97.3%) | 235 (97.9%) | 0.780 |  | 116 (95.9%) | 167 (98.8%) | 0.133 |  | 29 (90.6%) | 31 (91.2%) | 1.000 |
|  | Yes | 8 (2.7%) | 5 (2.1%) |  |  | 5 (4.1%) | 2 (1.2%) |  |  | 3 (9.4%) | 3 (8.8%) |  |
| TPS3 | No | 292 (100.0%) | 240 (100.0%) | 1.000 |  | 121 (100.0%) | 169 (100.0%) | 1.000 |  | 32 (100.0%) | 34 (100.0%) | 1.000 |
|  | Yes | 0 (0.0%) | 0 (0.0%) |  |  | 0 (0.0%) | 0 (0.0%) |  |  | 0 (0.0%) | 0 (0.0%) |  |
| PBRM1 | No | 209 (71.6%) | 189 (78.8%) | 0.071 |  | 117 (96.7%) | 162 (95.9%) | 0.767 |  | 31 (96.9%) | 34 (100.0%) | 0.485 |
|  | Yes | 83 (28.4%) | 51 (21.2%) |  |  | 4 (3.3%) | 7 (4.1%) |  |  | 1 (3.1%) | 0 (0.0%) |  |
| BAP1 | No | 275 (94.2%) | 222 (92.5%) | 0.484 |  | 115 (95.0%) | 162 (95.9%) | 0.780 |  | 32 (100.0%) | 34 (100.0%) | 1.000 |
|  | Yes | 17 (5.8%) | 18 (7.5%) |  |  | 6 (5.0%) | 7 (4.1%) |  |  | 0 (0.0%) | 0 (0.0%) |  |
| SETD2 | No | 273 (93.5%) | 220 (91.7%) | 0.504 |  | 113 (93.4%) | 162 (95.9%) | 0.423 |  | 32 (100.0%) | 33 (97.1%) | 1.000 |
|  | Yes | 19 (6.5%) | 20 (8.3%) |  |  | 8 (6.6%) | 7 (4.1%) |  |  | 0 (0.0%) | 1 (2.9%) |  |
| KDM5C | No | 283 (96.9%) | 231 (96.2%) | 0.811 |  | 119 (98.3%) | 169 (100.0%) | 0.173249 |  | 32 (100.0%) | 34 (100.0%) | 1.000 |
|  | Yes | 9 (3.1%) | 9 (3.8%) |  |  | 2 (1.7%) | 0 (0.0%) |  |  | 0 (0.0%) | 0 (0.0%) |  |
| KDM6A | No | 291 (99.7%) | 239 (99.6%) | 1.000 |  | 119 (98.3%) | 161 (95.3%) | 0.202 |  | 32 (100.0%) | 34 (100.0%) | 1.000 |
|  | Yes | 1 (0.3%) | 1 (0.4%) |  |  | 2 (1.7%) | 8 (4.7%) |  |  | 0 (0.0%) | 0 (0.0%) |  |
| mTOR | No | 292 (100.0%) | 240 (100.0%) | 1.000 |  | 121 (100.0%) | 169 (100.0%) | 1.000 |  | 32 (100.0%) | 34 (100.0%) | 1.000 |
|  | Yes | 0 (0.0%) | 0 (0.0%) |  |  | 0 (0.0%) | 0 (0.0%) |  |  | 0 (0.0%) | 0 (0.0%) |  |
| PIK3CA | No | 290 (99.3%) | 238 (99.2%) | 1.000 |  | 120 (99.2%) | 167 (98.8%) | 1.000 |  | 32 (100.0%) | 34 (100.0%) | 1.000 |
|  | Yes | 2 (0.7%) | 2 (0.8%) |  |  | 1 (0.8%) | 2 (1.2%) |  |  | 0 (0.0%) | 0 (0.0%) |  |
| TP53 | No | 288 (98.6%) | 235 (97.9%) | 0.738 |  | 120 (99.2%) | 164 (97.0%) | 0.406 |  | 25 (78.1%) | 21 (61.8%) | 0.185 |
|  | Yes | 4 (1.4%) | 5 (2.1%) |  |  | 1 (0.8%) | 5 (3.0%) |  |  | 7 (21.9%) | 13 (38.2%) |  |

*significant values
